# Preliminary Clinical Study of the Differences Between Interobserver Evaluation and Deep Convolutional Neural Network-Based Segmentation of Multiple Organs at Risk in CT Images of Lung Cancer

**DOI:** 10.3389/fonc.2019.00627

**Published:** 2019-07-05

**Authors:** Jinhan Zhu, Yimei Liu, Jun Zhang, Yixuan Wang, Lixin Chen

**Affiliations:** State Key Laboratory of Oncology in South China, Collaborative Innovation Center for Cancer Medicine, Sun Yat-sen University Cancer Center, Guangzhou, China

**Keywords:** contour variation, deep convolutional neural network, organs at risk, auto-contouring, lung cancer

## Abstract

**Background:** In this study, publicly datasets with organs at risk (OAR) structures were used as reference data to compare the differences of several observers. Convolutional neural network (CNN)-based auto-contouring was also used in the analysis. We evaluated the variations among observers and the effect of CNN-based auto-contouring in clinical applications.

**Materials and methods:** A total of 60 publicly available lung cancer CT with structures were used; 48 cases were used for training, and the other 12 cases were used for testing. The structures of the datasets were used as reference data. Three observers and a CNN-based program performed contouring for 12 testing cases, and the 3D dice similarity coefficient (DSC) and mean surface distance (MSD) were used to evaluate differences from the reference data. The three observers edited the CNN-based contours, and the results were compared to those of manual contouring. A value of P<0.05 was considered statistically significant.

**Results:** Compared to the reference data, no statistically significant differences were observed for the DSCs and MSDs among the manual contouring performed by the three observers at the same institution for the heart, esophagus, spinal cord, and left and right lungs. The 95% confidence interval (CI) and *P*-values of the CNN-based auto-contouring results comparing to the manual results for the heart, esophagus, spinal cord, and left and right lungs were as follows: the DSCs were CNN vs. A: 0.914~0.939(*P* = 0.004), 0.746~0.808(*P* = 0.002), 0.866~0.887(*P* = 0.136), 0.952~0.966(*P* = 0.158) and 0.960~0.972 (*P* = 0.136); CNN vs. B: 0.913~0.936 (*P* = 0.002), 0.745~0.807 (*P* = 0.005), 0.864~0.894 (*P* = 0.239), 0.952~0.964 (*P* = 0.308), and 0.959~0.971 (*P* = 0.272); and CNN vs. C: 0.912~0.933 (*P* = 0.004), 0.748~0.804(*P* = 0.002), 0.867~0.890 (*P* = 0.530), 0.952~0.964 (*P* = 0.308), and 0.958~0.970 (*P* = 0.480), respectively. The *P*-values of MSDs are similar to DSCs. The *P*-values of heart and esophagus is smaller than 0.05. No significant differences were found between the edited CNN-based auto-contouring results and the manual results.

**Conclusion:** For the spinal cord, both lungs, no statistically significant differences were found between CNN-based auto-contouring and manual contouring. Further modifications to contouring of the heart and esophagus are necessary. Overall, editing based on CNN-based auto-contouring can effectively shorten the contouring time without affecting the results. CNNs have considerable potential for automatic contouring applications.

## Introduction

The correct contouring of organs at risk (OARs) and target volumes is important for ensuring radiation quality during radiation treatment planning (RTP). Studies have shown that the dosimetric impact of the variation in the contouring of targets and OARs can be significant depending on the degree of variation and the plan dose gradient (1, 2). Differences in structure delineation impact DVH calculation, tumor control probability (TCP), and normal tissue complication probability (NTCP). The accuracy of primary gross tumor contouring could have a positive impact on tumor control and patient survival (3–5). Interobserver variation in the delineation of OARs primarily originates from various subjective interpretations of organ boundaries and objective contouring variation (6, 7). Reproducibility in the delineation of tumor and normal tissues is crucial for optimal treatment quality and outcomes (8). Variations in contouring have a direct impact on the quality and evaluation of RTP, especially for dose distribution of OARs (2). Intensity-modulated radiotherapy (IMRT) is a key treatment for lung cancer, particularly for patients with advanced stages (III and IV) (9). Cui Y et al. reported that the planned target volume (PTV) showed large variation among institutions. The PTV coverage of institutions dramatically decreased when re-evaluated using the consensus PTV contour (10). E.M. Gore et al. evaluated five thoracic radiation oncologists who collectively contoured cardiac structures for each available case, guided by a common atlas. The defined anatomic structures were the pericardium (P), ventricles (V), atria (A) and coronary spaces (CS). Large variation was found among observers, creating uncertainty regarding the dose delivered to OARs (11).

Standardized guidelines and anatomic atlases have been used to reduce interobserver variation and subjective diversity in clinical practice. The use of knowledge-based auto-contouring software, including atlas-based methods, has gained popularity because it is clinically acceptable, saves time and improves the consistency of contours created by various observers (1, 12–14). Rapid development has recently occurred for deep-learning methods, especially high-accuracy deep convolutional neural networks (CNNs), which can be used for computer vision, image recognition, and feature extraction (15–17). Neural networks are starting to be used for auxiliary diagnosis of medical images and contouring based on CT images (18, 19). Nevertheless, few studies have focused on the examination and comparison of the clinical use of neural networks regarding multiple OARs in CT images of lung cancer in RTP, particularly with respect to the following three questions: 1) Is there any difference between the results of CNN-based contouring and observer contouring? If so, which organs are different? 2) In clinical use, can interobserver variation and contouring time be reduced by editing the CNN-based auto-contouring results? 3) Based on these data, can CNN-based auto-contouring for OARs achieve an acceptable level for clinical use?

Datasets provided by the American Association of Physicists in Medicine (AAPM) in the thoracic auto-segmentation challenge were used as reference data. Variations among observers and the CNN and the clinical impact of editing based on CNN auto-contouring were evaluated.

## Materials and Methods

### Datasets

Publicly available lung cancer datasets were provided by AAPM for the thoracic auto-segmentation challenge in 2017 (20–22). The datasets were provided by three institutions: MD Anderson Cancer Center (MDACC), Memorial Sloan-Kettering Cancer Center (MSKCC) and the MAASTRO clinic. Each case had a CT volume and a reference contour. The contours were checked for quality and edited to adhere to the RTOG 1106 contouring guidelines (20). The OARs included heart, esophagus, spinal cord, left lung and right lung. Each image had 512 × 512 pixels and a layer thickness of 1.25–3 mm. There were 115–214 slices per case. The contours provided by the public datasets were used as the standards in the following analysis and the labeled data. A total of 60 cases were divided randomly into two groups, including a group of 48 cases for CNN training and a group of 12 cases for testing and evaluation.

### CNN-Based Auto-Contouring

A CNN is a specific type of multilevel perceptron architecture that can make predictions regarding an image. The largest difference between image contouring and image classification is that, in image contouring, the category of an object present in the image has to be identified, and the boundary of the object has to be depicted pixel by pixel (23, 24). The U–net architecture was first designed for biomedical image segmentation (25). The encoder gradually reduces the number of spatial dimensions and identifies the features of the image, while the decoder gradually modifies the details and spatial dimensions of the object and determines its boundary on a pixel-by-pixel basis. By considering that the volumes of the OARs are different in the thoracic region, DeepLabv3+ architecture combines the advantages of spatial pyramid pooling modules and encode-decoder structures. It uses atrous convolutions and atrous spatial pyramid pooling (ASPP) as the encoder for the segmentation of objects at multiple scales, and it uses a bilinear upsampling decoder module to refine the segmentation results, especially along the object boundaries (26). In the last layer, a 1 × 1 convolution with a softmax activation function reduces the number of feature maps to the number of labels.

### Image Preprocessing and CNN Training

To effectively increase the number of training samples, the training data were shuffled, and the following random processing tasks were performed during training: 1) each CT image was randomly cropped to regions of interest (ROIs) of 256 × 256 (columns × rows) pixels; the randomly cropped ROIs could overlap, but there was at least one labeled pixel in each ROI; and 2) the HU value was randomly shifted by ± 40 HU for each pixel.

To highlight soft tissue, bone, and spinal cord tissue, a window-level transformation was applied in which each original slice was transformed using a soft-tissue window (window width: 350; window level: 40), a bone window (window width: 1000; window level: 400), and a brain window (window width: 100; window level: 50) to generate three new images, and then, these images were integrated with the original image as an additional channel. The input size of the CNN was 256 × 256 × 4 (columns × rows × channels).

The training process requires automatic segmentation to be performed simultaneously for multiple organs that vary in size. Therefore, during the training process, the convergence rates vary. The class rebalancing properties of the generalized dice overlap, which is a recognized metric for segmentation assessment and a robust and accurate deep-learning loss function for unbalanced tasks (27). Adam optimizers were used to train the CNN, and the learning rate was 0.001. The following default values provided in the original paper were used for the other parameters: beta_1 = 0.9, beta_2 = 0.999, and epsilon = 1e−8 (28). The training batch size was 2, and the models were trained for 16 epochs.

### Interobserver Comparison of Contouring

To compare the differences among observers, 12 test cases were manually contoured by three observers. Observer A and observer B were experienced senior radiation oncologists specializing in the thoracic region with more than 10 years of work experience. Observer C was a dosimetrist with 6 years of work experience. The original structures of the test cases were deleted, and the three observers independently contoured the CT images using RTOG 1106 OAR contouring guidelines. Manual contouring was performed using Monaco (Elekta AB, Stockholm, Sweden). The observers were not shown the contours produced by the other observers.

Additionally, to evaluate whether the errors of the auto-contouring based on CNN lie into the variability of the experts, CNN-based auto-contouring was used as observer D and compared with the results of the three other observers.

Using the original structure of the test case as the reference data, the four contouring results (three manual and one automatic) were compared and analyzed in terms of the significant differences among the observers.

### Edited CNN-Based Contouring

The original structures of the test cases were deleted, and auto-contouring was performed on the test cases by the CNN. To minimize recall bias, the three observers independently reviewed and edited the final multisubject auto-contouring results of the OARs using consensus guidelines at a minimum of 1 month after manual contouring. The edited results were compared with the reference data for analysis.

### Quantitative and Statistical Analyses

Two indicators were used as evaluation criteria in the 3D region: the dice similarity coefficient (DSC) and the mean surface distance (MSD).

The DSC is commonly used to assess the degree of overlap between two structures in medical images (29). A higher level of overlap between two structures is reflected by a greater DSC. The DSC (0 ≤ DSC ≤ 1) is defined as follows:

(1) $$\begin{array}{r} DSC=\frac{2\left(V_{1}\;\cap \;V_{2}\right)}{V_{1}\;+\;V_{2}} \end{array}$$

where V_1_ is the volume of the reference structure and V_2_ is the volume of the comparison structure.

The formula for the MSD is:

(2) $$\begin{array}{r} d_{H,avg}\left(V_{1},\;V_{2}\right)=\frac{{\vec{d}}_{H,avg}\left(V_{1},\;V_{2}\right)+{\vec{d}}_{H,avg}\left(V_{2},\;V_{1}\right)}{2} \end{array}$$

where ${\vec{d}}_{H,avg}(V_{1},V_{2})=\frac{1}{\left|V_{1}\right|}\sum_{x\in V_{1}}\underset{y\in V_{2}}{\min}d(x,y)$, ${\vec{d}}_{H,avg}(V_{2},V_{1})=\frac{1}{\left|V_{2}\right|}\sum_{y\in V_{2}}\underset{x\in V_{1}}{\min}d(x,y)$, and x and y are points belonging to different structures. *d(x,y)* is the distance between x and y.

Statistical analysis was performed on the contouring results of the observers using the ranked Wilcoxon test. All analyses were performed using SPSS version 24.0 (SPSS, Chicago, IL, USA). A value of *P* < 0.05 was considered statistically significant.

## Results

### Comparison of Contouring Between Observers

Table 1 lists the 95% confidence interval (CI) and *P*-values for the statistical analysis of the DSCs for the OARs using pairwise comparisons among the observers. Except for the heart and esophagus, which were significantly different between observer D and observers A, B, and C (*P* < 0.05), no significant differences were found among observers for the other OARs. Table 2 lists the 95% CI and *P*-values for statistical analysis of the MSDs for OARs for pairwise comparisons among observers. Similar to the DSC results, significant differences were observed between observer D and observers A, B, and C only for the heart and esophagus (*P* < 0.05). The mean DSCs of the observers for the heart, esophagus, spinal cord, and left and right lungs met the commonly accepted threshold value for the DSC (DSC ≥ 0.7) (13, 14). The mean ± deviation of the DSC and MSD values for all observers compared to the reference data for the 12 test cases is listed in the Supplementary Material.

**Table 1:** 95% CI and *P*-value of Wilcoxon signed rank test comparing the DSC results generated by the individual observers.

	**Heart**		**Esophagus**		**Spinal cord**		**Lung_L**		**Lung_R**	
	**95% CI**	***P***	**95% CI**	***P***	**95% CI**	***P***	**95% CI**	***P***	**95% CI**	***P***
A vs. B	0.937~0.947	0.937	0.794~0.843	0.754	0.858~0.890	0.937	0.954~0.968	0.814	0.965~0.974	0.239
A vs. C	0.934~0.946	0.530	0.798~0.838	0.695	0.861~0.888	0.814	0.953~0.968	0.308	0.964~0.974	0.117
B vs. C	0.933~0.946	0.388	0.797~0.838	0.754	0.860~0.892	0.638	0.953~0.967	0.754	0.963~0.973	0.530
A vs. D	0.914-0.939	0.004	0.746-0.808	0.002	0.866-0.887	0.136	0.952-0.966	0.158	0.960-0.972	0.136
B vs. D	0.913-0.936	0.002	0.745-0.807	0.005	0.864-0.894	0.239	0.952-0.964	0.308	0.959-0.971	0.272
C vs. D	0.912-0.933	0.004	0.748-0.804	0.002	0.867-0.890	0.530	0.952~0.964	0.308	0.958-0.970	0.480

*A, B, and C are the three observers, D is the CNN-based auto-contouring*.

**Table 2:** 95% CI and *P*-value of Wilcoxon signed rank test comparing the MSD results generated by individual observers.

	**Heart**		**Esophagus**		**Spinal cord**		**Lung_L**		**Lung_R**	
	**95% CI**	**P**	**95% CI**	**P**	**95% CI**	**P**	**95% CI**	**P**	**95% CI**	**P**
A vs. B	1.41~1.75	0.937	0.85~1.64	0.480	0.67~0.85	0.638	0.95~1.52	0.754	0.98~1.52	0.754
A vs. C	1.50~1.93	0.182	0.91~1.55	0.480	0.72~0.87	0.433	0.99~1.55	0.480	1.02~1.50	0.209
B vs. C	1.52~1.96	0.158	0.91~1.56	0.272	0.68~0.88	0.530	1.03~1.53	0.308	1.02~1.50	0.480
A vs. D	1.78-2.79	0.003	1.19-1.88	0.034	0.72-0.85	0.530	1.02-1.79	0.071	1.07-1.73	0.158
B vs. D	1.81-2.81	0.002	1.19-1.89	0.034	0.68-0.86	0.937	1.06-1.78	0.433	1.08-1.73	0.272
C vs. D	1.96-2.93	0.010	1.26-1.79	0.019	0.73-0.88	0.583	1.10-1.81	0.875	1.10-1.71	0.272

*A, B and C are the three observers, D is the CNN-based auto-contouring*.

### Observer Editing of CNN-Based Contouring

Tables 3, 4 provide the 95% CI and *P*-values for the statistical analysis of the differences among manual contouring by the three observers and the edited contouring based on contours generated by the CNN. No statistically significant differences were found between independent manual contouring and the edited contouring for each OAR. However, the time required to edit the contours was reduced from 40–50 min to 15–20 min, effectively shortening the contouring time. The mean ± deviation of DSCs and MSDs for the CNN-based structures edited by the three observers with the reference data is listed in the Supplementary Material.

**Table 3:** 95% CI and *P*-value of Wilcoxon signed rank test comparing the DSCs generated by the manual method to those of the edited method for the individual observers.

	**Heart**		**Esophagus**		**Spinal cord**		**Lung_L**		**Lung_R**	
	**95% CI**	***P***	**95% CI**	***P***	**95% CI**	***P***	**95% CI**	***P***	**95% CI**	***P***
A	0.937~0.948	0.695	0.797~0.838	0.347	0.862~0.886	0.638	0.953~0.968	0.754	0.965~0.974	0.754
B	0.936~0.947	0.815	0.795~0.8374	0.638	0.865~0.8973	0.347	0.954~0.967	0.695	0.961~0.971	0.239
C	0.931~0.945	0.754	0.801~0.831	0.136	0.867~0.890	0.594	0.953~0.967	0.875	0.962~0.972	0.875

**Table 4:** 95% CI and *P*-value of Wilcoxon signed rank test comparing the MSDs generated by the manual method to those of the edited method for the individual observers.

	**Heart**		**Esophagus**		**Spinal cord**		**Lung_L**		**Lung_R**	
	**95% CI**	***P***	**95% CI**	***P***	**95% CI**	***P***	**95% CI**	***P***	**95% CI**	***P***
A	1.44~1.83	0.530	0.92~1.59	0.239	0.73~0.87	0.583	0.95~1.60	0.209	1.02~1.46	0.695
B	1.47~1.82	0.754	0.93~1.61	0.239	0.66~0.86	0.814	1.00~1.50	0.480	1.04~1.57	0.182
C	1.62~2.11	0.638	0.95~1.42	0.272	0.71~0.860	0.314	1.01~1.55	0.530	1.07~1.46	0.695

## Discussion

In this study, based on publicly available lung cancer datasets provided by AAPM, CNN-based auto-contouring was used as an observer (observer D) and compared to manual contouring performed by three separate observers. The differences among observers were analyzed for structures in publicly available datasets, which were used as the reference data. We found that, if the clinically acceptable level (DSC ≥ 0.7) was used as the standard (13, 14), the average DSCs of the heart, esophagus, spinal cord, and left and right lungs for the observers (including CNN auto-contouring) met the standard. However, for RTP, attention is focused on the difference in dosimetry parameters for various structures. Yunfeng Cui et al. (10) reported that, for non-small-cell lung cancer (NSCLC), the dosimetric impact of the variation of contouring OARs is dependent on the proximity of the OAR to the target and the dose gradient in the OAR region. OAR dosimetry was not highly affected by contouring in the observed variation range in their report.

For the spinal cord and left and right lungs, in the comparison with the reference data, the DSCs and MSDs were not significantly different between the results of CNN auto-contouring and the manual contouring of other observers because these three OARs have high contrast differences on CT, and their boundaries can be clearly identified. For the heart, most of the regional boundaries were clear on CT, and the average DSCs obtained by the four observers were >0.9. However, the boundaries of the starting and ending positions of the heart are not clear. The superior aspect begins at the level of the inferior aspect of the pulmonary artery. The HU value of the end position of the heart is close to those of the mediastinum and liver. Therefore, a significant difference was observed between CNN-based auto-contouring and manual contouring. Compared to those of manual contouring, the average DSC was reduced by 0.04 and the MSD was increased by 2.0 mm in CNN-based auto-contouring. Due to the poor soft-tissue contrast on CT images, the indistinct boundary of the esophagus due to surrounding soft tissues, and its irregular shape, both the DSCs and MSDs of CNN-based auto-contouring were significantly different from those of manual contouring. The average DSC was reduced by 0.08, and the MSD was increased by 0.59 mm. It is possible that the 48 cases used for training did not include patients with various esophageal shape changes and density variations. Therefore, the use of more uniform standard structures as training data may improve the results.

For the heart, esophagus, spinal cord, and left and right lungs, using the same standardized guidelines, no statistically significant differences were observed between the reference data and the three observers for the DSCs and MSDs. Dawn C. (30) reported that the magnitude of the discrepancies did not appear to be correlated with the experience of the dosimetrist for the heart, esophagus and spinal cord.

In the study, we found that using CNN-based contouring as a first pass for manual segmentation can increase the work efficiency. For RTP, precise delineation of OARs is a time-consuming process, especially because some OARs are difficult to differentiate from the other structures. On some CT slices, even experts have difficulties reliably defining boundaries (such as the esophagus), which leads to a tedious interpretation of CT findings and makes the process time-consuming and highly prone to interobserver variability. Some studies have shown that user editing of contours autogenerated by software is a viable strategy for reducing the contouring time of OARs while conforming to local clinical standards (18, 31). In this study, when editing CNN-based contours, the time could be reduced to 15–20 min on average. More importantly, no significant differences were found in the results of manual contouring and edited contouring. Therefore, adjustment of the results generated by a CNN can save the time required for OAR contouring while maintaining the accuracy and consistency of the contours. Nevertheless, the results presented in this study did not show that interobserver variation was reduced by editing CNN-based auto-contouring results. Unlike multi-institutional comparisons, the results presented in this study were generated by observers at the same institution who follow the same clinical contouring practices and have similar subjective interpretations of organ boundaries. Yunfeng Cui et al. (10) reported that a segmentation atlas improved the contour agreement for the esophagus and heart in a multi-institutional preclinical trial planning study. In a future study, multi-institutional observers should be included to determine the areas of agreement. Further investigation is needed to determine whether auto-contouring methods as described in this study could potentially reduce the interinstitutional observer variability for OARs.

This study is a preliminary clinical study on the examination and comparison of the clinical use of neural networks regarding multiple OARs in CT images of lung cancer in RTP. The total size of the data was limited to 60 cases, which were split for training and testing. The training data size would limit the CNN performance. However, assembling a large well-labeled dataset with consistent standards is very difficult. We hope to have higher quality data in the future. To effectively increase the number of training samples, the training data were shuffled, and random processing tasks were performed during training. These image generator preprocessing tasks can reduce the training difficulty caused by having too few samples, reduce model overfitting and increase the stability of the model. The results are statistically significant.

## Conclusions

In this study, publicly available lung cancer datasets were used as reference data. We compared and analyzed the differences between manual contouring by several observers and CNN-based auto-contouring for OARs. For the spinal cord and left and right lungs, no statistically significant differences were found between CNN-based auto-contouring and manual contouring. Further modifications to the heart and esophagus were necessary. Overall, editing CNN-based auto-contouring results can effectively shorten the contouring time while ensuring contouring quality.

## Author Contributions

All authors contributed to the research. JiZ completed the CNN program, performed the data analysis, and drafted the manuscript. LC developed the study concept, including the study design and coordination. YL, JuZ, and YW performed the manual contouring. All authors read and approved the final manuscript.

### Conflict of Interest Statement

The authors declare that the research was conducted in the absence of any commercial or financial relationships that could be construed as a potential conflict of interest.
