# Supplementary material for: Preliminary Clinical Study of the Differences Between Interobserver Evaluation and Deep Convolutional Neural Network-Based Segmentation of Multiple Organs at Risk in CT Images of Lung Cancer
Source: Front Oncol. 2019 Jul 5;9:627. doi: 10.3389/fonc.2019.00627 (PMC6624788; doi:10.3389/fonc.2019.00627)
Supplement: Supplementary file 1 [file Table_1.DOCX]

Supplementary Material

Preliminary clinical study of the differences between interobserver evaluation and deep convolutional neural network-based segmentation of multiple organs at risk in CT images of lung cancer

Jinhan Zhu, Yimei Liu, Jun Zhang, Yixuan Wang and Lixin Chen*

*** Correspondence:** Lixin Chen: chenlx@sysucc.org.cn

# Supplementary Tables

Table 1. Mean +/- deviation of DSCs of the individual observers compared to the reference data. Observer D is the CNN-based auto-contouring.

|  | Heart | Esophagus | Spinal cord | Lung_L | Lung_R |
| --- | --- | --- | --- | --- | --- |
| Observer A | 0.942±0.011 | 0.819±0.056 | 0.871±0.026 | 0.961±0.017 | 0.970±0.010 |
| Observer B | 0.942±0.014 | 0.818±0.057 | 0.877±0.044 | 0.960±0.015 | 0.969±0.010 |
| Observer C | 0.938±0.015 | 0.817±0.033 | 0.876±0.031 | 0.960±0.016 | 0.968±0.012 |
| Observer D | 0.907±0.024 | 0.735±0.061 | 0.882±0.020 | 0.956±0.013 | 0.961±0.015 |

Table 2. Mean +/- deviation of MSDs (mm) for the individual observers compared to the reference data. Observer D is the CNN-based auto-contouring.

|  | Heart | Esophagus | Spinal cord | Lung_L | Lung_R |
| --- | --- | --- | --- | --- | --- |
| Observer A | 1.56±0.37 | 1.24±0.92 | 0.78±0.16 | 1.23±0.71 | 1.25±0.63 |
| Observer B | 1.61±0.41 | 1.25±0.92 | 0.74±0.25 | 1.25±0.60 | 1.26±0.62 |
| Observer C | 1.87±0.56 | 1.22±0.53 | 0.82±0.19 | 1.33±0.56 | 1.27±0.49 |
| Observer D | 3.02±0.69 | 1.83±0.52 | 0.79±0.15 | 1.59±1.00 | 1.55±0.85 |

Table 3. Mean +/- deviation of DSCs of the individual observers generated by editing the CNN contouring compared to the reference data

|  | Heart | Esophagus | Spinal cord | Lung_L | Lung_R |
| --- | --- | --- | --- | --- | --- |
| Observer A | 0.943±0.013 | 0.816±0.040 | 0.877±0.030 | 0.960±0.017 | 0.968±0.010 |
| Observer B | 0.941±0.010 | 0.815±0.038 | 0.886±0.028 | 0.960±0.016 | 0.964±0.012 |
| Observer C | 0.938±0.015 | 0.815±0.035 | 0.883±0.021 | 0.961±0.016 | 0.967±0.012 |

Table 4. Mean +/- deviation of MSDs (mm) of the different observers generated by editing the CNN contouring compared to the reference data

|  | Heart | Esophagus | Spinal cord | Lung_L | Lung_R |
| --- | --- | --- | --- | --- | --- |
| Observer A | 1.71±0.53 | 1.26±0.61 | 0.82±0.18 | 1.32±0.78 | 1.24±0.37 |
| Observer B | 1.69±0.39 | 1.30±0.63 | 0.78±0.19 | 1.26±0.55 | 1.36±0.62 |
| Observer C | 1.86±0.58 | 1.13±0.56 | 0.75±0.13 | 1.25±0.66 | 1.27±0.41 |
